# A Mechanical Design Principle for Tissue Structure and Function in the Airway Tree

**DOI:** 10.1371/journal.pcbi.1003083

**Published:** 2013-05-30

**Authors:** Adam S. LaPrad, Kenneth R. Lutchen, Béla Suki

**Affiliations:** Department of Biomedical Engineering, Boston University, Boston, Massachusetts, United States of America; University of Virginia, United States of America

## Abstract

With every breath, the dynamically changing mechanical pressures must work in unison with the cells and soft tissue structures of the lung to permit air to efficiently traverse the airway tree and undergo gas exchange in the alveoli. The influence of mechanics on cell and tissue function is becoming apparent, raising the question: how does the airway tree co-exist within its mechanical environment to maintain normal cell function throughout its branching structure of diminishing dimensions? We introduce a new mechanical design principle for the conducting airway tree in which mechanotransduction at the level of cells is driven to orchestrate airway wall structural changes that can best maintain a preferred mechanical microenvironment. To support this principle, we report *in vitro* radius-transmural pressure relations for a range of airway radii obtained from healthy bovine lungs and model the data using a strain energy function together with a thick-walled cylinder description. From this framework, we estimate circumferential stresses and incremental Young's moduli throughout the airway tree. Our results indicate that the conducting airways consistently operate within a preferred mechanical homeostatic state, termed mechanical homeostasis, that is characterized by a narrow range of circumferential stresses and Young's moduli. This mechanical homeostatic state is maintained for all airways throughout the tree via airway wall dimensional and mechanical relationships. As a consequence, cells within the airway walls throughout the airway tree experience similar oscillatory strains during breathing that are much smaller than previously thought. Finally, we discuss the potential implications of how the maintenance of mechanical homeostasis, while facilitating healthy tissue-level alterations necessary for maturation, may lead to airway wall structural changes capable of chronic asthma.

## Author Summary

With every breath, mechanical pressures change in the lung and permit air to efficiently traverse the airway tree and undergo gas exchange. These pressure variations also influence cell and tissue function, raising the question: how does the airway tree co-exist within its mechanical environment to maintain normal cell function throughout its branching structure of diminishing dimensions? We introduce a new mechanical design principle for the conducting airway tree in which mechanotransduction, the process that converts mechanical forces on cells to biochemical signals, is driven to orchestrate tissue-level structural changes that can best restore a preferred mechanical microenvironment; a concept termed mechanical homeostasis. We report *in vitro* mechanical properties for a range of airway sizes and present a mathematical model that describes the data. Our results indicate that airways indeed consistently operate within a preferred mechanical homeostatic state. We further describe how this mechanical homeostasis while facilitating healthy tissue-level alterations necessary for maturation can inadvertently lead to airway wall structural changes capable of chronic asthma.

## Introduction

The act of breathing creates a mechanical environment that pervades all structures in the lungs down to the molecular level [1]. As a pressure difference across the lungs draws air through the airway tree for gas exchange, all airways dilate and transmit mechanical stresses and strains to their cellular constituents – including smooth muscle, epithelial, and fibroblast cells. All of these airway cell types reside within a complex bifurcating airway tree, and through mechanotransduction, they actively sense and respond to their mechanical environment [1]–[3]. Indeed, growth and remodeling of the extracellular matrix is consistently observed in response to chronically altered mechanical stress and occurs through a concerted response of multiple airway cell types [4]–[6].

This study asks the following question: What are the fundamental principles guiding the distribution of airway wall properties – in particular, airway wall thicknesses and airway wall material properties - throughout the conducting airway tree residing in a dynamic mechanical environment? Previous studies have examined airway tree design principles but have ignored both the dynamic mechanical forces to which the airway tree is perpetually subjected and the tissue-level biomechanical properties of the airway wall. Instead, based on the concept of a self-similar rigid tree [7] with optimal space-filling properties [8], it was presumed that the fractal design of airway luminal radii through the airway tree is optimal to transport fresh air to the periphery of the lung for efficient gas exchange. A tree design based purely on optimizing gas transport through rigid pipes ignores the fact that breathing dynamics can produce mechanically-driven alterations in the cells and tissue of the airway walls. Moreover, it is conceivable that these alterations would eventually modify the material properties and hence the caliber of the pipes themselves perhaps in a fashion destroying the underlying physical optimization related to gas transport [9].

Here, we introduce a new mechanically-based design principle for the airway walls of the conducting airways, in which mechanotransduction at the level of cells occurs in response to an altered mechanical microenvironment and is driven to orchestrate tissue-level structural changes of the airway wall to restore and maintain a preferred mechanical microenvironment. This theory is termed mechanical homeostasis and provides a plausible physiological role for mechanotransduction [10] in mechanically-driven tissue and organ systems. The concept has emerged as a prevalent theory in the vascular system [11]. However, mechanical homeostasis has not yet been conceptualized nor tested in the airway system.

Using experimental and modeling approaches, we show that the distribution of tissue-level biomechanical properties of the airway walls within the normal conducting airway tree is consistent with the existence of mechanical homeostasis. We also provide the likely desired homeostatic conditions for a healthy airway tree undergoing tidal breathing and occasional deep inspirations. Lastly, we address the implications of a mechanically-driven design principle with regard to airway disease. We conjecture that changes in the mechanical environment alone would facilitate healthy tissue-level alterations necessary for maturation on the one hand, but could also lead to airway wall structural changes capable of chronic asthma in a “misguided” attempt to sustain such mechanical homeostasis.

## Results

**Figure 1:**
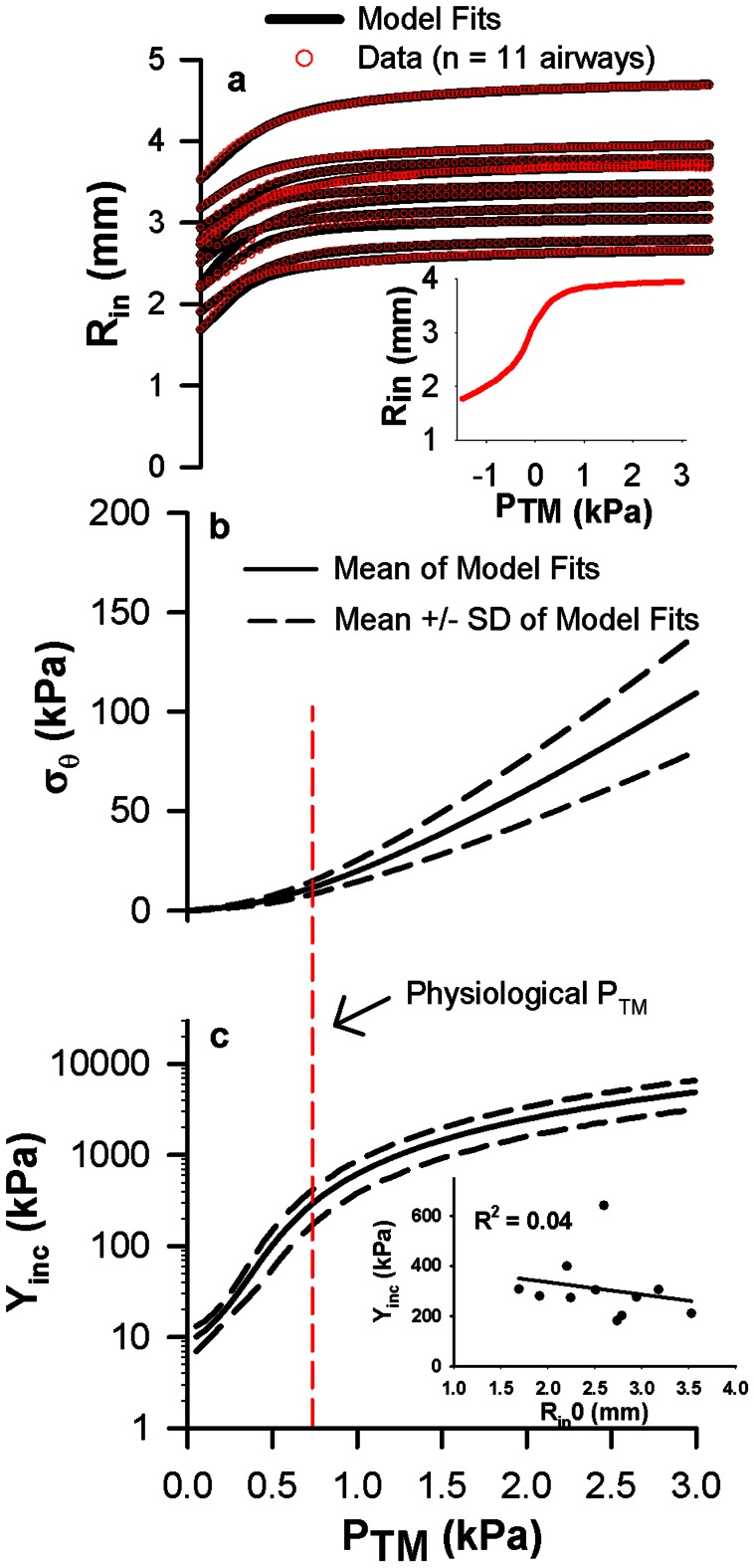
The existence of governing mechanical relationships in bovine intact airways. **a**, Relationships between luminal radius (R_in_) and transmural pressure (P_TM_) in 11 bovine airways. Red open circles are the measured data and black lines are the regression fits. The inset shows a representative example of a full R_in_ vs P_TM_ curve, from which we only utilize the positive pressures. **b**, Relationships between circumferential stress (σ_θ_) evaluated at the inner surface of the wall and P_TM_ calculated from the R_in_-P_TM_ regression analysis. **c**, Relationships between incremental elastic modulus (Y_inc_) and P_TM_ calculated from Eq. 6. In panels b and c, the black solid lines represent the mean of all 11 airways and the black dashed lines represent the mean +/− standard deviation. The red dashed line represents the typical operating P_TM_ experienced by airways. Note that the spread in σ_θ_ about the mean is small at the physiological P_TM_ of 0.75 kPa. The inset shows no correlation between Y_inc_ at 0.75 kPa P_TM_ and absolute airway size.

We first examine if and how the principles of mechanical homeostasis occur in structurally intact bovine airways *in vitro*. We measure the quasi-static relationships between airway luminal radius (R_in_) and transmural pressure (P_TM_) (Fig. 1a, red circles). By adopting a computational model of vascular mechanics using a strain-energy formulation for thick-walled cylindrical tubes [12], [13], we estimate the three-dimensional stresses and strains within intact airway wall tissue (Methods). We implement this analysis to determine the optimal model fits to our data for positive P_TM_ (Fig. 1a, solid lines), and calculate the relationships between the circumferential stress at the inner wall (σ_θ_) and P_TM_ (Fig. 1b, solid lines) and the corresponding incremental circumferential elastic modulus (Y_inc_) and P_TM_ (Fig. 1c, solid lines). This analysis identifies two salient mechanical features present within our airways. First, within the typical operating P_TM_ *in vivo* (0.5 to 1 kPa), every airway experiences a relatively narrow fixed range of σ_θ_ between about 8 and 15 kPa, which is less than 6% of the maximum stress. We approximate this as a fixed circumferential stress of 12 kPa at the mean operating P_TM_ of 0.75 kPa in Fig. 1b. Second, for all 11 airways measured, there is a nearly identical relationship between Y_inc_ and P_TM_ with values between 180 and 310 kPa at P_TM_ = 0.75 kPa, as defined by the dashed curves in Fig. 1c. Importantly, we find no correlations between Y_inc_ at 0.75 kPa P_TM_ and absolute airway size (Fig. 1c inset) suggesting that the airway wall stiffness is approximately the same for many generations of the airway tree when evaluated around the physiological operating P_TM_ of 0.75 kPa.

**Figure 2:**
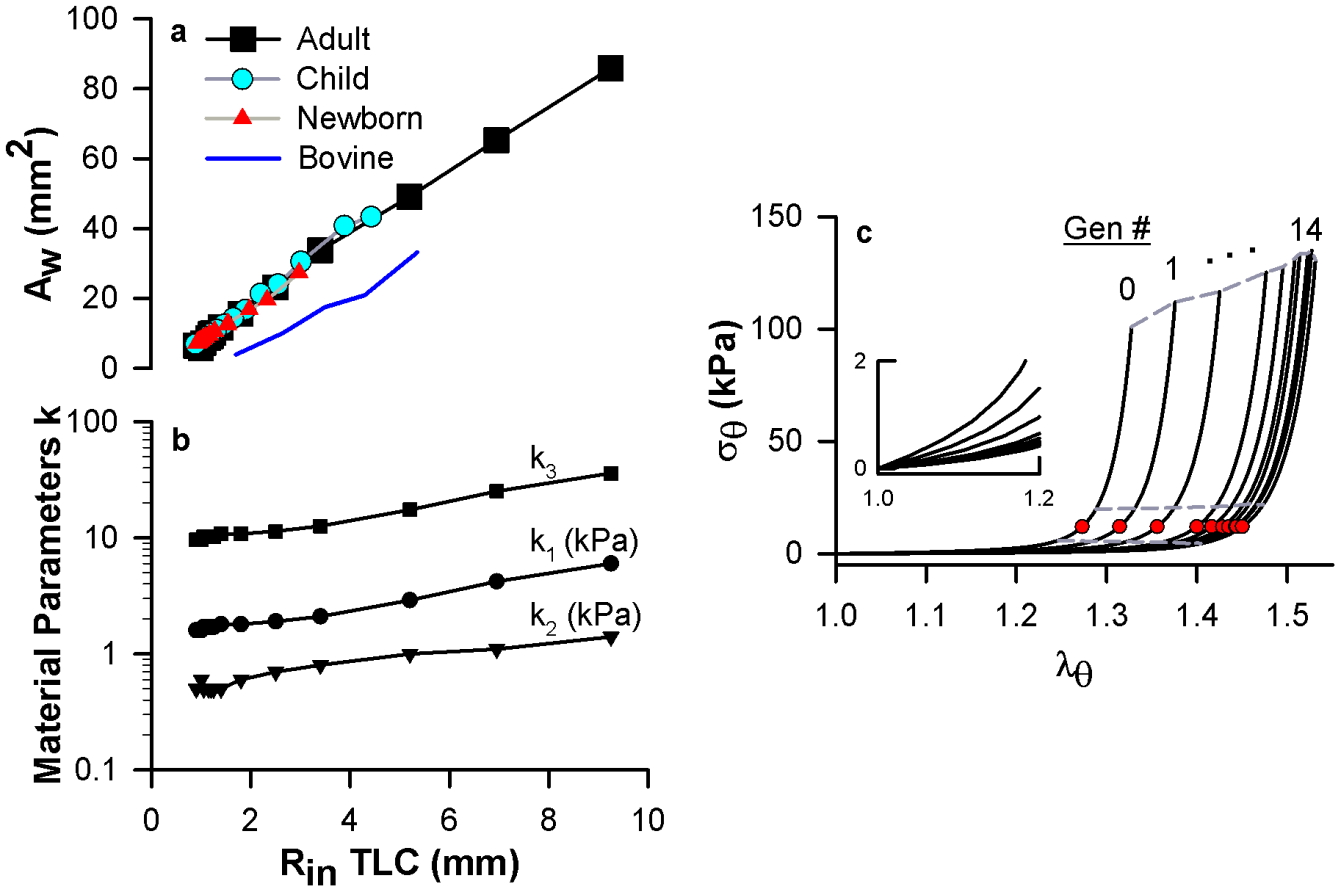
Mechanical homeostasis is maintained throughout the airway tree by a combined generation-dependence in wall dimensions and model-derived material parameters. **a**, Relationship between mean airway wall area (A_w_) and mean luminal radius (R_in_ TLC) for healthy humans obtained and reanalysed from the literature: newborns [15] (red triangles, 0 year old), children [15] (cyan circles, 3 years old), and adults [14] (black squares, mean age of 39.2 years old). The corresponding relationship for our bovine airways is shown in blue. Circles represent individual airway generations, where the largest radius in each data set corresponds to the trachea (generation 0). All dimensions are measured at total lung capacity (TLC, 2.5–3.0 kPa P_TM_). **b**, The model-predicted relationship between the material parameters k_1_, k_2_, k_3_ and the luminal radius at TLC (R_in_ TLC). **c**, The model-predicted generation dependence in circumferential stress (σ_θ_) vs. circumferential stretch ratio (λ_θ_) from generation 0 to generation 14. Red circles are the corresponding stress-stretch at 0.75 kPa P_TM_. Dashed lines are the corresponding stress-stretch at 0.5, 1.0, and 3.0 kPa P_TM_. The inset is an enlargement of the range from 0 to 1.2 λ_θ_.

We next utilize a computational modeling approach to examine how the uniform σ_θ_ and Y_inc_ would be maintained throughout an airway tree structure exposed to the transmural pressures of breathing. The substantial decreases in luminal radius from the trachea (generation 0) to the periphery (generation 26) presumably allow for optimal gas transport via fractal branching but would also result in vastly different circumferential stresses for the resident cells within the airways, as evident in simplistic terms by the geometric relationship of LaPlace's law (σ_θ_ = P_TM_ * R_in_/H where H = wall thickness). To maintain a constant σ_θ_ and Y_inc_ at the operating P_TM_ throughout the airway tree, the airway wall areas and material properties would need to play a compensatory role within the thick-walled cylindrical airway. We re-analyze high resolution computed tomography (HRCT) data measured in humans by several investigators [14], [15] (Methods) to discover a strong linear relation between airway wall area and airway luminal radius throughout the airway tree (Fig. 2a). Remarkably, this relationship is maintained throughout all stages of lung growth from birth to adulthood. That is, an airway at the periphery of an adult lung, which has the same luminal radius as a central airway in a child, also has the same wall area. Taken together with our results of radius-independent micromechanical environment in Fig. 1, these findings are consistent with the existence of mechanical homeostasis within the airway tree.

Using the geometric relationship in Fig. 2a, we next predict the airway wall material parameters required to maintain the data-derived conditions of mechanical homeostasis (from Fig. 1) throughout the airway tree. By necessity, the model-determined material parameters describing the nonlinear elasticity of the airway wall (Eq. 1) increase from the periphery to the trachea (Fig. 2b). From the generation-dependent geometric (Fig. 2a) and material (Fig. 2b) relationships, we then compute σ_θ_ and the circumferential stretch ratio (λ_θ_) (Fig. 2c, solid lines) for the entire airway tree and consequently, we obtain Y_inc_ (Fig. 2c, slopes of solid lines). The peripheral airways have smaller Y_inc_ than the central airways at any given λ_θ_, which is consistent with observed decreases in collagen and cartilage content down the airway tree [16]. As a direct consequence, the progressively increasing stretch ratios along the nonlinear σ_θ_- λ_θ_ curves allow the peripheral airways to experience the same σ_θ_ and Y_inc_ as the central airways when exposed to typical operating P_TM_ (Fig. 2c, cyan circles).

**Figure 3:**
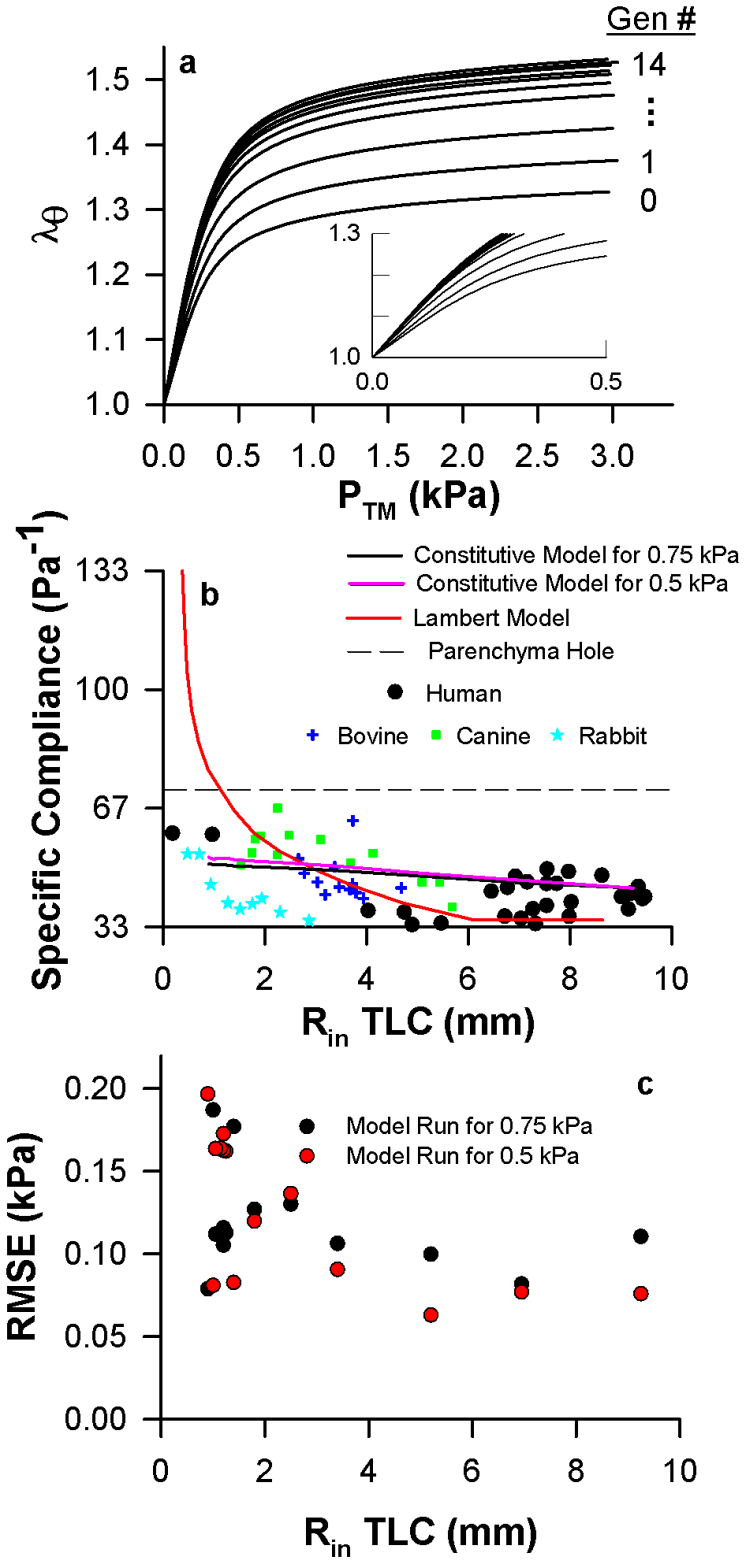
The generation-dependence in airway wall compliance is a direct consequence of homeostatic circumferential stresses and incremental elastic moduli throughout the airway tree. **a**, The relationship between circumferential stretch ratio (λ_θ_) and transmural pressure (P_TM_) for all generations. The inset is an enlargement of the range from 0 to 0.5 kPa P_TM_. **b**, The relationship between specific airway compliance from 0 to 3.0 kPa P_TM_ and the luminal radius at TLC for our analysis at both 0.75 operating P_TM_ (black line) and 0.5 kPa operating P_TM_ (magenta line) and reanalysis and plotting a previous model by Lambert et al. [22] (red line). Expansion of a parenchymal hole (dashed line) was calculated assuming that lung volume at 0 kPa P_TM_ is 15% of volume at 3.0 kPa as shown in data [22]. For comparison, our bovine data is shown (blue crosses) along with data visualized from the literature in humans [17]–[19] (black circles), dogs [20] (green squares), and rabbits [21] (cyan stars). **c**, The root mean square errors (RMSE) between the model and data assuming optimal P_TM_ of 0.5 kPa or 0.75 kPa as a function of airway radius.

To validate our model prediction, we utilize the predicted σ_θ_- λ_θ_ curves to calculate the relationships between λ_θ_ and P_TM_ for the airway tree (Fig. 3a). These relationships are directly measurable in human lungs *in situ* for large airways [17] (greater than 2 mm luminal radius) and *in vitro* for peripheral human airways [18], [19]. From the trachea to the periphery, our analysis predicts that the airways are progressively more compliant. The specific airway compliance (Δλ_θ_/ΔP_TM_) from 0 to 3 kPa P_TM_ modestly increases from the trachea (4.4 Pa^−1^) to the periphery (5.1 Pa^−1^) (Fig. 3b, solid black line). Our predictions of specific airway compliance are consistent with the human data in the literature [17]–[19] (Fig. 3b, black circles), our bovine data (Fig. 3b, blue crosses), and data in dogs [20] and rabbits [21] (Fig. 3b, green squares and cyan stars). Importantly, our predictions are also consistently below the estimate of parenchymal hole expansion, which is generally accepted as the limit of airway expansion (Fig. 3b, dashed line). We also performed a sensitivity analysis that shows that these predictions remain essentially the same when the calculations are repeated for an optimal P_TM_ of 0.5 kPa (solid magenta) instead of 0.75 kPa (solid black). Additionally, the root mean square error (RMSE) between model and data are similar for both P_TM_ of 0.5 kPa and 0.75 kPa independent of airway radius (Fig. 3c). Thus, the agreement between our model predictions and the data in Fig. 3b is consistent with the notion that the known generation-dependence in specific airway compliance exists as a means to maintain a constant intrinsic mechanical microenvironment in response to P_TM_ throughout the airway tree.

**Figure 4:**
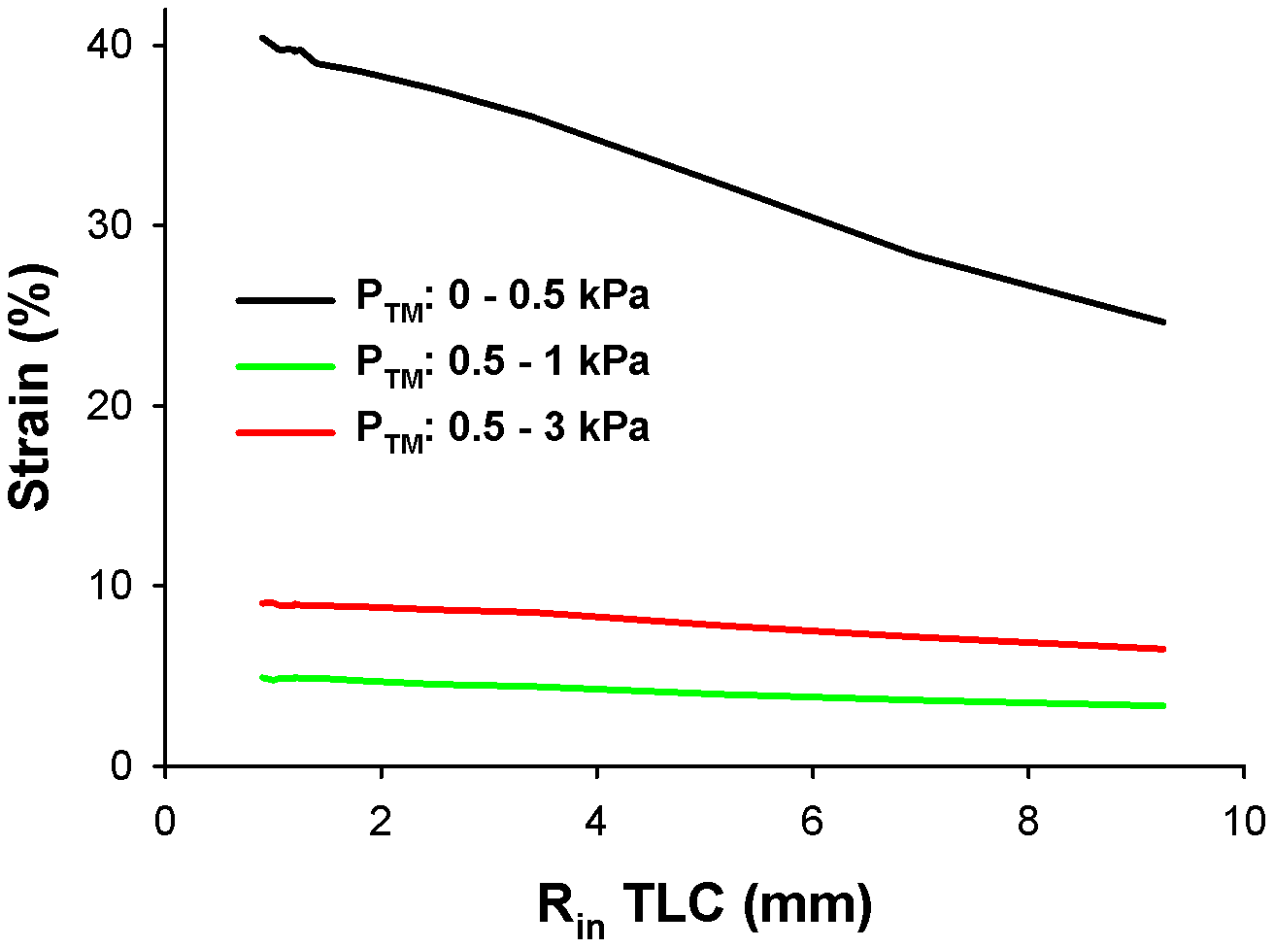
Distribution of circumferential strain throughout the airway tree. Strain was calculated as the relative % difference in airway radius at the inner wall, R_in_, for various changes in P_TM_ (strain = [R_in_(P_TM_2)−R_in_(P_TM_1)]/R_in_(P_TM_1) * 100%). Black line represents the strain corresponding to inflating an un-pressurized airway to P_TM_ = 0.5 kPa, approximately functional residual capacity. The green line and the red line represent the predicted strains during tidal breathing between 0.5 and 1 kPa P_TM_ and deep inspiration between 0.5 and 3 kPa P_TM_, respectively.

With only modest increases in specific airway compliance down the tree, our results also suggest that cells in the airway wall are well-equipped to respond uniformly to strain-dependent phenomena, such as cellular fluidization from deep inspirations [3]. Since it has not been possible to directly measure small airway mechanical properties *in vivo*, computational models of airway wall mechanics are vital to estimate the mechanical strains of breathing that are used in mechanobiological cell culture and tissue strip experiments. One prevailing model developed by Lambert et al. [22] was based on the extrapolation of limited empirical fits of radius-P_TM_ data [17]. This model has been used in isolated ASM strips to apply *in vivo* like loads that would mimic a small airway's structure (1.1 mm luminal radius). In this experimental model, deep inspirations to TLC result in large sustained reductions in ASM constriction [23]. These results are in stark contrast to P_TM_ oscillations applied directly to constricted bovine [24] and human [25] airways, which have little to no impact on airway caliber. Interestingly, we find that while the Lambert model has similar predictions to our model for large airways, the Lambert model also predicts airways having unrealistically large compliance as radius decreases (Fig. 3b, solid gray line), which eventually become much larger than the available data and the parenchymal hole expansion limit. In contrast, our model suggests that the mechanical strains experienced by ASM cells during breathing are much smaller than previously thought as our simulations demonstrate (Fig. 4). Furthermore, these strains depend only mildly on airway size increasing from 3.3% and 6.5% at the trachea to 4.9% and 9% in small airways during tidal breathing and deep inspiration, respectively. As a consequence, their sustained functional impact on airway responsiveness would be greatly attenuated when tested under physiologically appropriate mechanical conditions [24], [25].

## Discussion

Our data and modeling analyses suggest that the organ-level structure of the airway system plays a crucial role in sustaining not just organ level function, namely gas transport, but also the function of its individual components down to the cellular level in every airway wall throughout the airway tree during breathing. Specifically, the airway tree's structural design maintains circumferential stresses and circumferential elastic moduli in a relatively narrow range. The circumferential stress and the incremental modulus determine the effective stiffness of the extracellular matrix which is known to significantly influence cellular behavior [2]. Thus, the regulation of the mechanical microenvironment throughout the airway tree (Fig. 1), during growth (Fig. 2a), and in response to tidal breathing (Fig. 4) to maintain a homeostatic state ensures normal cellular and tissue-level function within a dynamic P_TM_ environment in a way that maintains compatibility with the physical structure of the airway tree required for optimal gas transport. It is interesting to note that while homeostasis is formulated in terms of stress and modulus, it also results in a nearly constant circumferential strain independent of airway radius (Fig. 4) implying that similar cells in the walls but with different sensitivity to strain did not have to evolve for efficient airway function. Thus, the consistency of tissue-level structure with a mechanical design principle further implies an active and essential role of airway cells as controllers of mechanical homeostasis for the airway wall system. While airway luminal radius and length have evolved to efficiently deliver gas through a tree structure [8], the airway wall tissue structure has evolved to provide the proper mechanical milieu for its constituent cells.

Before examining the implications of a mechanical design principle, we first discuss the limitations of our intact airway experiments and computational modeling approaches. We used bovine intact airways *in vitro* to obtain pressure-radius relationships. Compared to tissue strips and cell culture, this preparation maintains the 3-dimensional architecture of the airway and applies a physiological transmural pressure as a means of stretching the cells and the extracellular structures in the airway wall. However, neural airway tone that is known to constantly modulate airway diameter *in vivo* [26] is removed in this preparation. When using these data in our computational approach, we neglected the pressure-diameter relation for negative P_TM,_ which may be important during flow limitation; we found that to describe the negative P_TM_ data, we would need a different form of the strain energy function. While our analysis of the data advances airway wall modeling by invoking a thick-walled cylinder approach, the model uses several simplifying assumptions. We assume that the airway walls are elastic, which is a limitation of the strain energy density formalism itself. We also assume that the airway walls are homogeneous and maintain a cylindrical shape at all P_TM_. During breathing, the lung is exposed to cyclic stretch and the irregular nature of the stretch pattern has important consequences on cellular function [27]. If cellular growth or remodeling of the airway are of interest, these irregular stretch patterns may influence these processes. The thick-walled hollow cylinder is assumed to be homogeneous and isotropic, which are the usual assumptions in airway wall modeling. At the length scale of single cells, however, this is certainly not true. We also assume that the typical operating pressure is 0.75 kPa for all conducting airways and for all stages of life (birth to adulthood). It is probable that the operating pressure may change throughout the course of development, and it may also differ slightly throughout the airway tree due to gravitational differences in pleural pressure, resistive losses in airway pressure down the airway tree, surface tension, and local differences in parenchymal tethering forces. A sensitivity analysis, however, revealed that almost identical results are obtained for operating pressures of 0.75 kPa and 0.5 kPa.

Another limiting assumption in both our data analysis and computational approach is plane strain, in which the properties and deformation of the airway wall are assumed to not change in the axial direction. In our *in-vitro* experiments, the two ends of the airways are fixed at a pre-stretch ratio that is consistent with in-vivo lengthening that occurs during tidal breathing [28]. In this setup, the plane strain condition is indeed valid for a section of the airway in the middle away from the boundaries which we verified using the ultrasound; data only from this region are used in the analysis. However, in the *in-vitro* experiments and in our computational approach, airways cannot lengthen dynamically with radial expansion, as presumably occur *in-vivo*. Relaxing the plane strain assumption to allow for airway lengthening would require complex finite element modeling and knowing the *in vivo* boundary conditions for the airway which are beyond the scope of this study.

**Figure 5:**
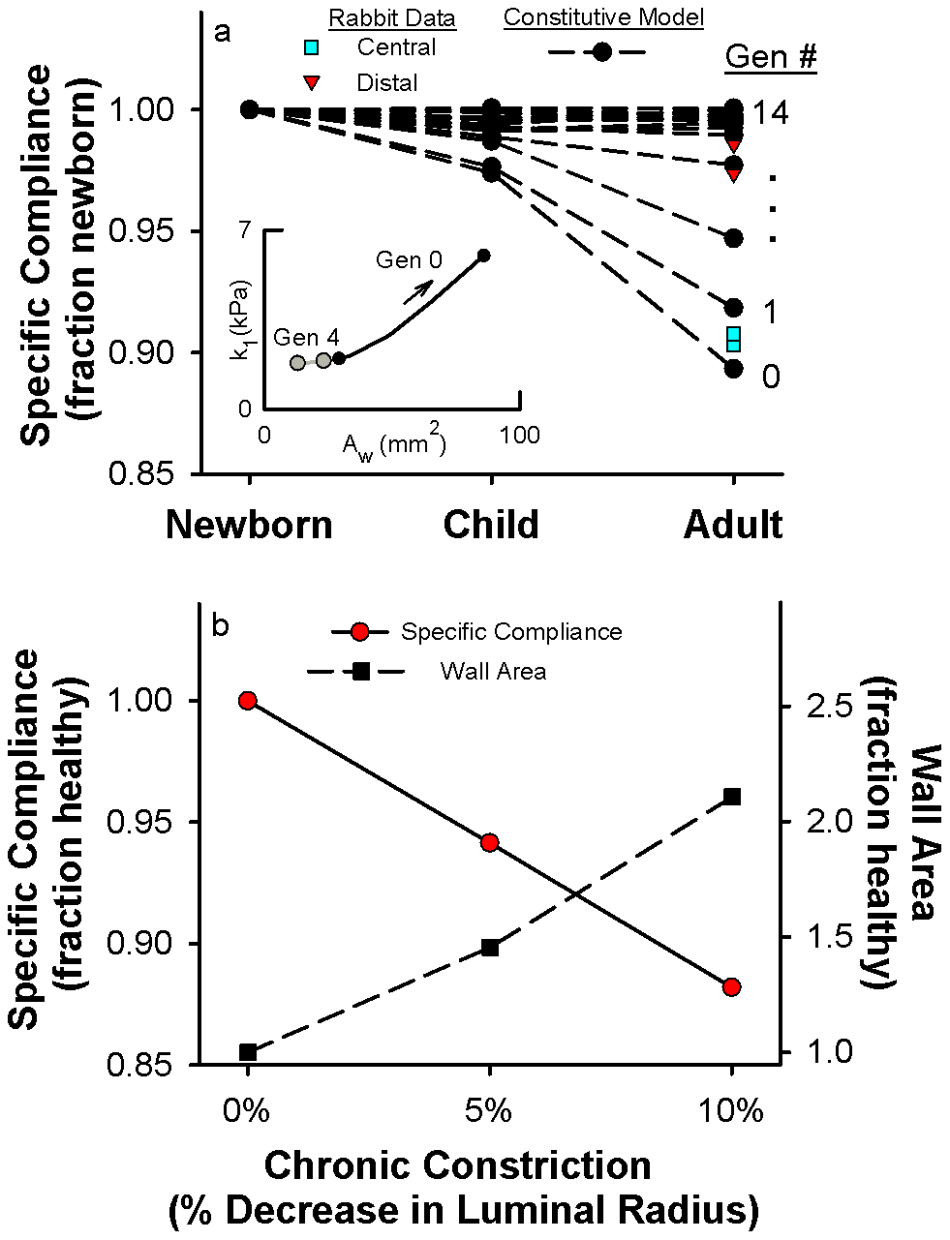
The principles of mechanical homeostasis provide a framework for the processes of airway wall growth during lung development and airway wall remodelling in asthma. **a**, Relationship between the specific airway compliance from 0 to 3.0 kPa P_TM_ and stages of lung development as predicted by the model (black circles) and measured in central and distal airways of rabbits [21] (cyan squares and red triangles, respectively). The specific airway compliance for each generation is normalized to its value at birth (newborn). The inset shows the model-predicted increases in wall area and material parameter k_1_ from a newborn to an adult for a generation 0 airway (black circles) compared to a generation 4 airway (gray circles). **b**, Fractional changes in the specific airway compliance from 0 to 3.0 kPa (red circles, solid line) and wall area (black squares, dashed line) due to levels of chronic constriction, represented as a percentage decrease in airway luminal radius. The calculations were performed for a generation 0 airway after removal of ASM stimulation (e.g., a simulated bronchodilator), thus representing the sustained structural remodelling.

We next discuss the implications of mechanical homeostasis on the behavior of airways over various time scales. On short time scales, ASM cells can actively control local stresses in the wall by contraction and relaxation. This has indeed been observed in dogs where airway lumen varied substantially from day-to-day over a period of a year [29]. On longer time scales, many other cell types participate in controlling stresses via remodeling of the airway wall. In fact, the consistency of geometric dimensional relationships from birth to maturity in the airway tree (see Fig. 2a) suggests that proper tissue growth is not a pure biochemical process but it also requires significant mechanically-driven feedback. As growth factors cause an airway's luminal radius to increase in size [30], the circumferential stress and elastic modulus would also initially increase at a given operating P_TM_. However, cellular compensatory mechanisms would detect this deviation from mechanical homeostasis and trigger the airway cells to build more wall tissue to restore the homeostatic state, characterized by an optimal circumferential stress and incremental Young's modulus. Therefore, a generation 0 airway at birth would grow to a much larger luminal radius in maturity while maintaining a similar preferred mechanical environment. As a consequence of this airway wall growth process, our analysis predicts a generation-dependent decrease in airway wall compliance as the airways mature (Fig. 5a, black circles), which is in agreement with data in the literature [21] (Fig. 5a, squares and triangles). This proposed process is consistent with an emerging hypothesis that the unique geometric and material properties of mature organs derive from mechanical stimuli and feedback throughout development [31] and may add another mechanical stimulus for growth in the respiratory system [32].

In an analogous fashion, our results also have implications for airway diseases. While the airway wall structure is designed to maintain normal cell function under physiological operating conditions throughout life, it remains ill-prepared for persistent ASM activation from environmental sources. ASM contraction immediately changes airway wall circumferential stresses by introducing an active mechanical stress that reduces airway caliber and increases wall thickness. Thus, airway cells would be immediately driven from their physiologic mechanical homeostatic state. Over time, repetitive ASM activation would stimulate airway wall remodeling processes consistent with growth in an attempt to restore mechanical homeostasis. Chronic constriction would result in an airway wall that is thicker and stiffer (Fig. 5b), as is consistent with data on asthmatic airways in the literature [33]. However, as ASM activation is sporadic in nature and occurs on a much faster timescale than structural remodeling, the presence of airway remodeling indicates a tissue-level system struggling to restore a steady-state mechanical homeostasis that may never be fully realized. Applied throughout the airway tree, this deviation from mechanical homeostasis would have disastrous impacts to function at the level of the cell, tissue, organ, and organism [6], [9] and would not be readily reversible with bronchodilators [33]. Mechanical homeostasis may thus emerge as a governing principle that divides health and disease within the respiratory system, and may also unify respiratory diseases with a host of others whose progression is intimately coupled to mechanical signaling [34].

## Methods

### Ethics statement

Experiments were carried out using bovine lungs obtained from a local slaughterhouse immediately after death (Research 87, Bolyston, MA). Protocol approval was not required.

### Measurement of pressure-radius relationships in excised bovine airways

Our system for intact airway experiments has been described previously [35]. Briefly, bovine lungs were obtained from a local slaughterhouse. A bronchus of the right lung (generations 10–17) was dissected and the side branches were ligated. The airway was cannulated at each end and mounted horizontally in a tissue bath containing gassed (95% O2-5% CO2) and heated (37°C) Krebs solution. The airway was stretched longitudinally (110–120% of its resting length) and held fixed at its extended length for the entire experiment. Tissue viability was then confirmed with both electric field stimulation and acetylcholine (ACh; 10 5 M).

A computer-driven pressure-controlled syringe pump delivered P_TM_ changes to the intact airway. The proximal cannula was mounted in series to a hydrostatic pressure column filled with Krebs solution, which also filled the airway lumen. The difference in fluid height between the horizontally mounted airway and the pressure column determined the intraluminal pressure (thus P_TM_) experienced by the airway.

A portable ultrasound system (Terason 2000), consisting of a high-frequency linear array transducer (10L5) and an external beamformer module, was used to visualize the intact airway. The hardware was connected directly to a personal computer running software that both controlled the imaging settings (focal depth, focal length, and gain) and acquired and stored the images in real time. The ultrasound transducer was mounted above the intact airway and partially submerged in the tissue bath. Using a three-directional micromanipulator, the transducer was positioned over the airway's longitudinal axis at its diameter. The airway was imaged at 30 fps with fixed ultrasound imaging settings (focal depth: 30 mm, focal length: 13 mm, gain: 0.2).

We controlled P_in_ delivered to the structurally intact bovine airways of fixed length while R_in_ and R_out_ were directly measured with ultrasound imaging. Quasi-static, passive R-P_TM_ curves were measured via slow ramps in P_in_ (−1.5 to 3 kPa, 0.1 kPa/second) with P_out_ set to 0 kPa. The positive expiratory limb (3 to 0 kPa) was used for our analysis to probe the full physiological range of breathing (0.5 kPa P_TM_ at functional residual capacity to 3 kPa at total lung capacity). Eleven bovine airways were obtained from the right lower lobes of eight different animals.

#### Mathematical framework to characterize the mechanical properties of intact airways

As with blood vessels, airways experience large deformations and exhibit nonlinear mechanical properties, both of which are not accounted for in simple constitutive relationships based on the assumptions of linear elasticity and infinitesimal strains. For several decades, the field of arterial wall mechanics has evolved to manage these complexities through the development of finite-deformation analysis based on the framework of nonlinear continuum mechanics [12]. In this framework, a hyperelastic material is defined by a strain energy density function (SEDF), from which it is postulated that the stress in the material can be obtained by the derivative of the SEDF with respect to strain. Numerous functional forms of SEDFs have been developed for blood vessels and have been shown to fit experimental data well [12], [13]; this mathematical framework has allowed for a deeper understanding of vascular structure-function relationships in health and disease [11], but it has yet to be applied to airways in a rigorous manner.

Guided from the previously developed vascular SEDFs [12], [13], we arrived at the following functional form to best describe our airway wall tissue mechanical properties. Specifically, we found that airways require a highly nonlinear SEDF to describe the noncompliant regime – as shown by the plateau of the radius vs. pressure curve (Fig. 1a) – at large luminal pressures.
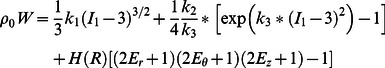
(1)Our analysis assumes that the airway wall is hyperelastic, incompressible, homogeneous, isotropic, and undergoes plane strain. The parameters k_1_, k_2_, k_3_ represent the material properties of the airway wall tissue. The Lagrangian multiplier H(R) enforces the condition of incompressibility. The first strain invariant I_1_ was calculated from the stretch ratio components in the circumferential (λ_θ_), radial (λ_r_), and axial (λ_z_) directions:
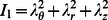


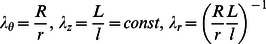


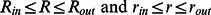
The radius R represented the airway in the deformed (axially stretched and pressurized) state and the corresponding radius r represented the airway in its undeformed state. Under the plane strain assumption, the axial stretch ratio was fixed. L represented the deformed fixed length and the corresponding length l represented the undeformed length.

From the SEDF, the Cauchy stresses 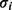 for i = r, θ, z were calculated as:
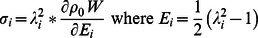
As in arterial wall mechanics [12], [13], we modelled the airway as a thick-walled cylindrical tube. The equilibrium equation has been derived elsewhere [36] and, in final form, is:
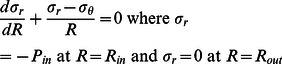
(2)From the equilibrium equation (Eq. 2), one can obtain the following constitutive equations relating normal stresses in the circumferential (σ_θ_), radial (σ_r_), and axial directions (σ_z_) to stretch ratios as derived elsewhere [13], [37]:
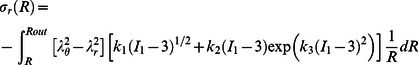
(3)

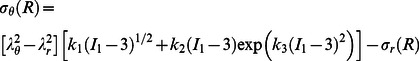
(4)

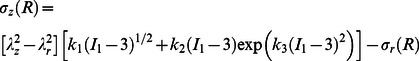
(5)Lastly, the incremental elastic modulus (Y_inc_) in the circumferential direction was computed as:
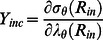
(6)


#### Estimation of material parameters from intact airway data

In an intact airway *in vitro*, the following boundary condition can be invoked to estimate the material parameters k_1_, k_2_, k_3_ from measured luminal pressure (P_in_), luminal radius (R_in_), and outer radius (R_out_):
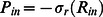
(7)For the measured R-P_in_ data, a grid search algorithm was used to determine the material parameters k_1_, k_2_, k_3_ that minimized the sum of squared errors between the measured P_in_ and model predicted P_in_ using Eqs. 3 and 7.

#### Analysis of human airway wall dimensions from literature

The adult airway wall dimensions were obtained directly from a data set in the literature [14]. The toddler and newborn dimensions were calculated from a set of regressions between airway dimensions and body length for the first four generations (0–3) measured in children less than 3 years old [15]. To express airway dimensions as a function of age, we determined the linear regression between age and body length (R^2^ = 0.87). We defined a child as 3 years old and a newborn as 0 year old. The adult, child, and newborn data sets all had an exponential decay in luminal radius with generation from which we extrapolated the luminal radii for generations 4–14 in the toddler and newborn. Finally, using the linear relationship between wall area and luminal radius (Fig. 2a), we extrapolated wall areas for generations 4–14. Both studies used multi-detector computed tomography imaging to measure airway dimensions *in vivo* [14], [15].

#### Predictions of airway wall properties throughout the airway tree

We assumed all airways in the tree have the same functional form of the SEDF, as previously described. From our bovine airway analysis (Fig. 1a–c), we defined two conditions of mechanical homeostasis to apply throughout an airway tree. First, there is an optimal fixed relationship between Y_inc_ and P_TM_ for every airway as defined by the mean curve of Fig. 1c (black solid line). Second, at an airway's typical operating P_TM_, the circumferential stress has a fixed value as defined by the mean value at 0.75 kPa P_TM_ in Fig. 1b. We then prescribed the human adult airway dimensions from the literature [14] in Fig. 2a (luminal radius and wall area) and assumed a constant axial pre-stretch ratio (1.2).

Using Eqs. 4 and 6, we determine the material parameters needed to fulfil the data-derived conditions of mechanical homeostasis and the airway dimensions of human adults for each generation of the conducting airways. A grid search algorithm was used to determine the optimal material parameters k_1_, k_2,_ k_3_ by minimizing the sum of the root mean squared errors between the two prescribed conditions from above and the model predicted relationships of Y_inc_ vs. P_TM_ and σ_θ_ at 0.75 kPa P_TM_. In this analysis, k_1_ controls the airway distensibility for 0<P_TM_<1 kPa (governed structurally by elastin) and k_3_ controls the maximum distensibility at 3 kPa (governed structurally by collagen). For this reason, we assumed that the two material parameters are directly related (k_3_ = 6k_1_). We also assumed that all airways are exposed to the same transmural pressure under typical operating conditions (0.75 kPa). Thus, we disregarded gravitational differences in pleural pressure, resistive losses in airway pressure down the airway tree, surface tension, and local differences in parenchymal tethering forces.

#### Predictions of airway wall properties during airway growth and chronic constriction

Using the airway wall dimensions for newborns, children, and adults (Fig. 2a), we calculated the change in specific airway wall compliance that would occur as airways at each airway generation grow from birth to maturity while maintaining the data-derived conditions of mechanical homeostasis. To do this, we assumed that the relationship between material parameters and wall area derived in the previous section (Figs 2a and 2b) is also the same relationship that governs airway growth, resulting in the growth profile of Fig. 4a, inset. Next, to arrive at Fig. 4a, we used Fig. 2a to prescribe airway luminal radius and wall area for newborns, children, and adults at each airway generation and then used Fig. 4a, inset to determine the corresponding material parameters from the wall area. With values for the luminal radius, wall area, and material parameters, we then calculated the radius-pressure curves using Eqs. 3 and 6 and calculated the specific airway compliance from the radius-pressure curves.

Lastly, we determined how airways held chronically at smaller radii due to ASM contraction would remodel to maintain circumferential stress homeostasis. If an adult airway with a healthy homeostatic stress-stretch relationship (from Fig. 2c) is constricted due to ASM contraction, the airway would experience a circumferential stress that is smaller than its homeostatic value at a fixed transmural pressure of 0.75 kPa, for instance. To re-establish mechanical homeostasis at this reduced radius, the airway wall material parameters and wall area would need to change governed by active cellular remodeling. In Fig. 4b, we determined the remodelled material parameters and wall area needed to restore the homeostatic circumferential stress value while following the growth profile of Fig. 4a, inset. To compare how the passive mechanical properties had changed due to the remodelling alone (and not because of simply moving down the radius-pressure curve with ASM activation), we removed the chronic constriction (e.g., with a simulated bronchodilator) and calculated the passive specific compliance (Fig. 4b).
